# Post-pandemic changes in anxiety and depression symptom networks among socioeconomically disadvantaged young Adults: A repeated cross-sectional study

**DOI:** 10.1016/j.ssmph.2025.101854

**Published:** 2025-08-16

**Authors:** Aziz Essadek, Tamara Guenoun, Florence Gressier, Maha Najdini, Maud Cappelletti, Antoine Frigaux, Maria Melchior, Maeva Musso, Marion Robin

**Affiliations:** a Laboratoire INTERPSY UR4432, Université de Lorraine, 54015 Nancy, France; b Hopital Saint-Maurice, Paris, France; c Laboratoire CRPPC, Université de Lyon, Lyon, France; d CESP, INSERM U1018, Moods Team, Faculté de Médecine Paris Saclay, University Paris-Saclay, Le Kremlin Bicêtre, 94275, France; e Centre Psychothérapique de Nancy, 1 Rue Dr Archambault, Laxou, 54520, France; f Sorbonne Université, INSERM, Institut Pierre Louis d'Epidémiologie et de Santé Publique (IPLESP), Equipe de Recherche en Epidémiologie Sociale (ERES), Cedex 12, Paris, 75571, France; g Department of Adolescent and Young Adult Psychiatry, Institut Mutualiste Montsouris, 75014 Paris, France

## Abstract

**Background:**

The COVID-19 pandemic has significantly affected the mental health of young adults, particularly those facing socioeconomic hardship. Although psychological distress appears to be declining in the general population post-pandemic, vulnerable subgroups remain at elevated risk. Network analysis offers a transdiagnostic approach to understanding the dynamic interplay of depressive and anxiety symptoms over time.

**Methods:**

We conducted a repeated cross-sectional study among socioeconomically disadvantaged young adults in 2020 (T1) and 2024–2025 (T2). Depressive and anxiety symptoms were assessed using the PHQ-9 and GAD-7. Symptom networks were estimated using Gaussian Graphical Models with EBICglasso. The Network Comparison Test (NCT) evaluated changes in network structure and symptom centrality. Clustering analysis was performed to explore the reorganization of symptom groupings over time.

**Results:**

Mean scores increased significantly between T1 (n = 960) and T2 (n = 380) for both depression (PHQ-9: 9.43 to 11.35, *p* < 0.001) and anxiety (GAD-7: 6.3 to 8.14, *p* < 0.001). Suicidal ideation nearly doubled (25.9 %–42.9 %, *p* < 0.001). Network analysis revealed stronger interconnections between depressive and anxiety symptoms at T2. Anxiety symptoms (particularly GAD3, GAD2, and GAD1) became more central, while suicidal ideation shifted from a depression-specific cluster to one integrating anxiety symptoms. Clustering analysis supported a progressive integration of depressive and anxiety domains.

**Conclusion:**

Our findings suggest an evolving post-pandemic psychopathological network, with anxiety symptoms becoming increasingly central and closely linked to suicidal ideation. These results underscore the need for targeted interventions addressing both depression and anxiety, particularly among socioeconomically vulnerable young adults, to more effectively reduce suicide risk in this population.

## Highlights

• Disadvantaged young adults' mental health worsened post-pandemic, more depression, anxiety, and doubled suicidal ideation.
•Symptom network analysis reveals a major shift, with anxiety symptoms becoming more central than depressive symptoms.
•Suicidal ideation is now closely linked to anxiety symptoms, suggesting that anxiety plays a direct role in suicide risk.
• Findings urge rethinking prevention, focusing on anxiety disorders, especially among the most vulnerable youth.

The mental health of young adults has emerged as a major public health concern since the onset of the COVID-19 pandemic. Prior to the health crisis, the prevalence rates of depressive and anxiety disorders among this population ranged from 6.1 % to 8.3 % in the United States (Han et al., 2018), with a concerning rate of suicide attempts reaching 14 % (Piscopo et al., 2016). From the early waves of the pandemic, studies have consistently reported a sharp increase in depression, anxiety, and suicidal ideation worldwide (Essadek et al., 2022; Wathelet et al., 2022; Xiong et al., 2020), with prevalence rates estimated to be up to three times higher than those observed in the pre-pandemic period (Ettman et al., 2020).

The decline in mental health has been particularly pronounced among young adults, largely due to lockdown measures, social isolation, and economic instability, all of which have contributed to the deterioration of their psychological well-being. This increased vulnerability may also be explained by the specific developmental needs of this age group, including the crucial importance of social connections, autonomy, and social integration (Essadek et al., 2023; Huh et al., 2023; Liang et al., 2022; Wathelet et al., 2022). Since the lifting of pandemic-related restrictions, several cross-sectional and longitudinal studies have reported a gradual decrease in anxiety and depression levels, as well as a reduction in suicidal ideation in the general population (Reutter et al., 2024). However, this overall improvement does not indicate a return to baseline, and certain vulnerable subgroups continue to exhibit high levels of psychological distress, or even worsening conditions (Kiviruusu et al., 2024). The post-pandemic increase in psychiatric care utilization and psychotropic medication prescriptions (Fond et al., 2025) suggests that, despite an apparent improvement in mental health indicators, certain subgroups, particularly young adults and women (Fond et al., 2025), emain at heightened risk.

A longitudinal study conducted in Germany (Ahrens et al., 2021) revealed that the impact of lockdown was not uniform. While most participants maintained or regained good mental health, 8.4 % exhibited delayed deterioration, highlighting a phenomenon that remains underexplored: certain negative effects of the pandemic may surface well after the lifting of restrictions. These findings underscore the need for a differentiated approach across subgroups to better understand the diversity of post-pandemic psychological trajectories.

In this context, young adults facing socioeconomic hardship appear particularly at risk (Wathelet et al., 2022). Several studies suggest that financial insecurity (Chan et al., 2024) food insecurity (Essadek et al., 2023), and employment instability (Ervin et al., 2024), have played a key role in the decline of mental health within this population. However, most research has focused on general or student populations, leaving more vulnerable subgroups underexamined, despite the added complexity of their care during this period (Payan et al., 2024).

To examine the psychological consequences of the pandemic on socioeconomically disadvantaged populations, we adopted a network analysis within a transdiagnostic framework. Rather than focusing on discrete diagnostic categories, this framework emphasizes shared psychological processes and symptom interactions across disorders. Network analysis is particularly suited to this approach, as it enables the exploration of the structure and dynamics of mental disorders by identifying central symptom nodes that may play a key role in the persistence or progression of anxiety and depressive states (Borsboom, 2017). Using this approach, Bai et al. (Bai et al., 2021) demonstrated that irritability, uncontrollable worry, and depressed mood were central symptoms in the psychopathological network among university students, suggesting priority targets for intervention. Similarly, Fico et al. (Fico et al., 2023) found that although social support and resilience have protective effects, they remain insufficient to mitigate pandemic-related mental health disorders.

Furthermore, Ochnik et al. (Ochnik et al., 2024) provided insights into the temporal evolution of depressive and anxiety symptom networks by comparing two distinct phases of the pandemic. Their findings showed a persistent increase in depression, with depressed mood (PHQ2 - Sad Mood) emerging as the central symptom, and highlighted a shift in bridging symptoms over time: initially dominated by suicidal ideation at June 2020 (T1), the network later centered around anhedonia at March 2021 (T2). A key contribution of this study lies in the identification of protective factors, particularly life satisfaction and physical activity, which appear to mitigate the severity of depressive and anxiety symptoms.

Beyond examining the evolution of mental health from the period of acute psychological distress to the current situation, our study aims to analyze the dynamics of depressive and anxiety symptom networks among young adults experiencing socioeconomic precarity. By adopting a repeated cross-sectional design, we seek to identify persistent central symptoms as well as factors that modulate these psychopathological trajectories. This population is of particular interest because socioeconomically disadvantaged young adults face a cumulative burden of vulnerability, including housing instability, social exclusion, and limited access to care. Rather than excluding these already at-risk individuals, focusing on them allows us to better understand how large-scale crises like the COVID-19 pandemic may amplify existing disparities in mental health. By integrating both the temporal evolution of disorders and the underlying maintenance mechanisms, this analysis will contribute to a more nuanced understanding of post-pandemic psychological vulnerabilities and help inform more targeted interventions for high-risk populations.

## 1 Method

### 1.1 Data source

This study is based on a repeated cross-sectional design conducted among young adults experiencing socioeconomic precarity and enrolled in the vocational integration program of the Paris *Mission Locale*. This program supports individuals aged 16 to 25, although only participants aged 18 and older were included in the present study. It offers individualized support including career counseling, access to training, and coordination with social services. Data collection occurred at two time points, four years apart. The first wave (T1) was conducted in December 2020, one week after the peak in COVID-19-related mortality during the second lockdown in France. The second wave (T2) took place between December 2024 and March 2025. The active file of the Paris Mission Locale comprises approximately 9000 young people per year. Based on this population, a minimum sample size of 368 participants was estimated using classical survey methodology, assuming a 95 % confidence level and a 5 % margin of error. However, the actual number of participants reflects the total number of young adults who voluntarily completed the questionnaire during each wave, as the study was conducted in real-world conditions using a non-probabilistic convenience sampling method. In the context of network analysis, the required sample size depends not only on representativeness but also on the stability of the estimated parameters. Recent methodological literature recommends a minimum of 300–500 participants for reliable estimation of symptom networks involving 15–20 nodes, especially when interpreting centrality indices or comparing networks over time (Epskamp et al., 2018a; Bringmann et al., 2019). At T1 (N = 960), the sample size exceeded this threshold, allowing for robust estimation of symptom structures and network metrics. At T2 (N = 380), the sample size remained within the acceptable range for estimating network structures, although caution is warranted when interpreting local differences in connectivity. The difference in sample sizes between T1 and T2 reflects contextual and organizational variations across the two data collection periods.

The survey was administered online using LimeSurvey. Participants were invited to take part via text message and email, which included a direct link to the questionnaire. All participants provided informed consent prior to completing the survey. To ensure confidentiality and data integrity, responses were anonymized, and participants were able to pause and resume the survey at any time.

### 1.2 Measures

The study collected socio-demographic data to characterize young adults in socioeconomic precariousness in both cohorts. Information included age, gender, level of education, housing type and employment status, and living in isolation. Economic indicators were also considered, including access to financial support and income level (no resource, <€500, €500–1000, >€1000).

Psychological symptoms were assessed using validated scales. Depression was measured using the Patient Health Questionnaire-9 (PHQ-9; range: 0–27) (Kroenke et al., 2001), with a score of ≥10 indicating clinically significant symptoms (Manea et al., 2012). Anxiety was assessed using the Generalized Anxiety Disorder scale (GAD-7; range: 0–21) (Spitzer et al., 2006), with a threshold of ≥8 identifying clinically relevant anxiety (Plummer et al., 2016). Suicidal ideation was evaluated using item 9 of the PHQ-9, which assesses the presence and frequency of suicidal thoughts (Rossom et al., 2017). For this study, responses with missing data were excluded from the analyses.

### 1.3 Statistical analysis

First, we computed descriptive statistics for each sample corresponding to the two study waves: 2020 (T1) and 2024–2025 (T2). Quantitative variables were expressed as means and standard deviations (SD), while qualitative variables were reported as percentages (%). The chi-square (χ^2^) test of independence was used to examine whether the distribution of sociodemographic variables significantly differed between the two samples. Student's t-test was applied to compare the mean age of participants. Effect size was assessed using Cohen's d, with the following thresholds: small (d ≥ 0.20), medium (d ≥ 0.50), and large (d ≥ 0.80). Socioeconomic variables that significantly differed between T1 and T2 (e.g., housing status and income) were included as covariates in the regression models to adjust for potential confounding effects. Adjusted odds ratios for depression, anxiety, and suicidal ideation were subsequently computed, controlling for age, gender, education level, housing status, and income. Internal consistency was high for both the PHQ-9 and GAD-7 scales at each time point. For the PHQ-9, Cronbach's alpha was 0.85 at T1 and 0.89 at T2. For the GAD-7, it was 0.89 at T1 and increased slightly to 0.90 at T2. These values indicate good to excellent reliability across both samples, supporting the robustness of the symptom measures over time.

#### 1.3.1 Network analysis

To identify symptom interactions and their evolution between T1 and T2, we first estimated Gaussian Graphical Models (GGMs) separately for each network. As the Shapiro–Wilk test indicated that not all variables followed a normal distribution, a nonparanormal (NPN) transformation was applied (Liu et al., 2012) (see Supplementary Data Fig. 1, Fig. 2). This transformation approximates multivariate normality while preserving monotonic relationships between variables. Correlations were calculated using Spearman's rank coefficient on the transformed data to ensure a robust estimation of the underlying relational structure. We used the EBICglasso method to estimate the networks. This approach promotes model parsimony by reducing non-significant connections and minimizing the risk of spurious associations. The networks were visualized using a spring layout, which arranges nodes (symptoms) based on the strength of their connections. Edges represent partial correlations between symptoms, with their thickness reflecting the strength of the relationships.

**Fig. 1:**
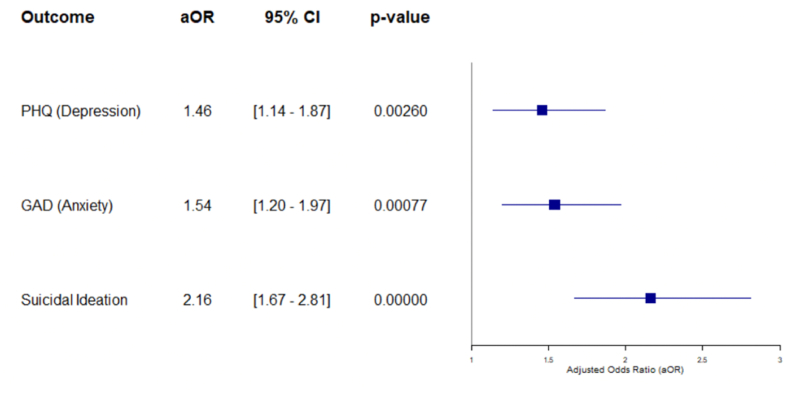
Forest Plot of adjusted Odds Ratio (aOR).

**Fig. 2:**
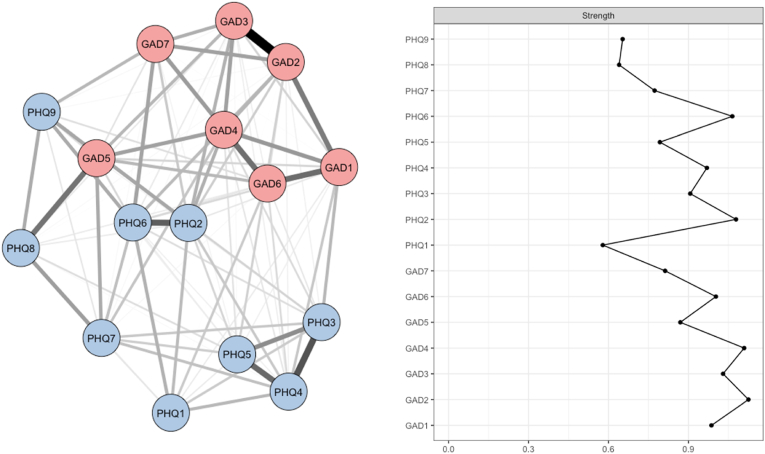
Network analysis at T1. **Legend:** The figure on the left displays the network analysis of depression (PHQ-9) and anxiety (GAD-7) symptoms at T1. The figure on the right shows strength centrality values for individual items of the PHQ-9 (depression) and GAD-7 (anxiety) scales. PHQ-9 items include: PHQ1 = Anhedonia, PHQ2 = Sad Mood, PHQ3 = Sleep, PHQ4 = Energy, PHQ5 = Appetite, PHQ6 = Worthlessness, PHQ7 = Concentration, PHQ8 = Psychomotor, PHQ9 = Suicidal Ideation; GAD-7 items include: GAD1 = Nervousness, GAD2 = Uncontrollable Worry, GAD3 = Excessive Worry, GAD4 = Trouble Relaxing, GAD5 = Restlessness, GAD6 = Irritability, GAD7 = Fear of Awful Events.

#### 1.3.2 Centrality and network stability analysis

The stability of centrality indices was assessed using case-dropping bootstrapping (n = 1000), in which increasing proportions of the sample were removed and the centrality metrics re-estimated. A network is considered stable when its centrality indices remain consistent despite data removal. Robustness was quantified using the correlation stability coefficient (CS-C) (Epskamp et al., 2018a). A CS-C value above 0.25 is considered acceptable, with values above 0.5 being preferable (Epskamp et al., 2018b). Centrality refers to how important or influential a given symptom (node) is within the overall symptom network. It reflects the extent to which a symptom is connected to others and may contribute to the maintenance or escalation of psychological distress. In our study, we evaluated several centrality metrics, including strength, betweenness, and closeness. However, consistent with prior research indicating that closeness and betweenness may be unstable and less reliable (Bringmann et al., 2019; Epskamp et al., 2017), we primarily interpreted node strength, which is the most robust and widely used index in network analysis (Haslbeck & Waldorp, 2018). Additionally, we examined node predictability to measure the degree of variance explained by their direct neighbors. Our findings support these prior observations, with node strength demonstrating greater stability compared to other centrality indices.

#### 1.3.3 Network comparison between T1 and T2

To examine the evolution of symptom networks between T1 and T2, we employed the Network Comparison Test (NCT), a non-parametric approach based on 1000 permutations. This method enables the assessment of changes in network structure and symptom connectivity over time. Network structure invariance was tested to determine whether the pattern of connections between symptoms differed across time points, under the null hypothesis of structural stability. Global strength invariance was analyzed to evaluate whether the overall intensity of association between nodes had changed. We also examined differences in symptom centrality, focusing exclusively on node strength. p-values were interpreted to identify any statistically significant differences.

#### 1.3.4 Symptom clustering analysis

To complement the Network Comparison Test (NCT) and further explore the evolution of symptom relationships between T1 and T2, a clustering analysis was conducted. This approach allows for the identification of groups of highly interconnected symptoms and the examination of their stability over time. First, the symptom networks were converted into graphs and analyzed using the Louvain method, which detects communities by optimizing cluster modularity. This method groups symptoms based on the strength of their interconnections, providing a complementary view of the symptom structures identified through NCT. Next, hierarchical clustering was performed using Euclidean distances computed from Spearman correlation matrices. The Ward.D2 algorithm was applied to minimize within-cluster variance and enhance the coherence of symptom groupings. The robustness of the clusters was evaluated using bootstrapping (n = 1000), which allowed us to assess the stability of symptom groupings at T1 and T2. Cluster composition was compared across the two time points to identify potential structural changes. These analyses refine the interpretation of the NCT results by highlighting symptom reorganization over time.

#### 1.3.5 Statistical packages

All analyses were conducted using R (version 4.4.2). Network estimation and visualization were performed using the *qgraph* and *bootnet* packages (Epskamp et al., 2012, 2018b), while comparisons between T1 and T2 were carried out using the *NetworkComparisonTest* package (Van Borkulo et al., 2023). Clustering analyses were conducted using the *cluster* and *pvclust* packages (Suzuki & Shimodaira, 2006). The *factoextra* package was used to visualize dendrograms.

The study received ethical approval from the Ethics Committee of the University of Lorraine (AE2025-0037) and the Ethics Committee of the Hôpitaux de Saint-Maurice (PR-2024-6).

## 2 Results

### 2.1 Sociodemographic variable

The sample included 960 participants at T1 and 380 at T2. The mean age was slightly higher at T2 than at T1, with a statistically significant but small difference (d = −0.13, p = 0.032). Although the proportion of women was similar across the two time points (49.5 % at T1 vs. 49.7 % at T2), a statistically significant but small difference was observed (p = 0.022, V = 0.07). Finally, although a statistically significant difference was found between T1 and T2 regarding financial resources, indication a slight increase over time, the effect size was small (p = 0.001, V = 0.13). Additional sociodemographic variables are presented in Supplementary Data, Table 1.

### 2.2 Mental health variables

Between T1 and T2, a significant increase in psychiatric symptoms was observed. The mean PHQ-9 score, increased from 9.43 [SD = 6.7] to 11.35 [SD = 7.2] (t = −4.47, p < 0.001), while the mean GAD-7 score, rose from 6.3 [SD = 5.9] to 8.14 [SD = 6.2] (t = −4.95, p < 0.001) (see Supplementary Data, Table 1). The proportion of participants reporting moderate to severe depression (PHQ-9 ≥ 10) increased from 46.9 % to 56.3 % (p = 0.002), and those with clinically significant anxiety symptoms (GAD-7 ≥ 8) rose from 36.7 % to 47.9 % (p < 0.001). Suicidal ideation also increased, from 25.9 % to 42.9 % (p < 0.001). Adjusted odds ratio analyses confirmed these trends: the risk of depression was 1.46 times higher at T2 (aOR = 1.46, 95 % CI [1.14–1.87], p = 0.003); the risk of anxiety increased by a factor of 1.54 (aOR = 1.54, 95 % CI [1.20–1.97], p < 0.001); and the risk of suicidal ideation more than doubled (aOR = 2.16, 95 % CI [1.67–2.81], p < 0.001) (see Fig. 1 & Supplementary Data, Table 2).

### 2.3 Network analysis at T1

The analysis of relationships between PHQ-9 and GAD-7 symptoms at T1 reveals a well-structured symptom organization (Fig. 2). The network comprises sixteen nodes and displays moderate connectivity, with 94 non-zero edges out of 120 possible, indicating a marked degree of symptom interdependence. The average edge strength was 0.17, reflecting meaningful interactions between certain symptoms without excessive connectivity. The network architecture shows distinct subgroups, with depressive and anxiety symptoms interconnected while still retaining their specific clustering. Among the strongest associations, robust connections were observed between GAD1 (Nervousness) and GAD2 (Uncontrollable) (0.38), PHQ6 (Worthlessness) and PHQ8 (Psychomotor) (0.35), and PHQ3(Sleep) and PHQ4 (Energy) (0.32) (Supplementary Data, Fig. 1). Centrality analysis identified key symptoms within the network's structure. At T1, PHQ8 (psychomotor retardation) exhibited the highest centrality score (0.78), suggesting it plays a pivotal role in the depressive symptom network. On the anxiety side, GAD1 (worry) and GAD2 (nervous tension) had centrality scores of 0.65 and 0.63, respectively, forming a stable core within the anxiety network. The average edge weight was estimated at 0.21 (95 % CI [0.14–0.35]), confirming relatively strong connections among certain central symptoms. Bootstrap-based stability estimation yielded a mean coefficient of 0.84, ensuring the robustness of the identified network structure. The network configuration revealed a concentration of connections around the most central nodes, underscoring their structuring role. The centrality strength coefficient (CS) further confirmed this organization, with high values for GAD1 (Nervousness), GAD2 (Uncontrollable), and PHQ8 (psychomotor retardation), consolidating their pivotal role within the symptom network (supplementary data, Fig. 3). These findings suggest a coherent symptom organization at T1, with distinct but interconnected clusters, reflecting the complexity of interactions between depressive and anxiety symptoms.

### 2.4 Network analysis at T2

**Fig. 3:**
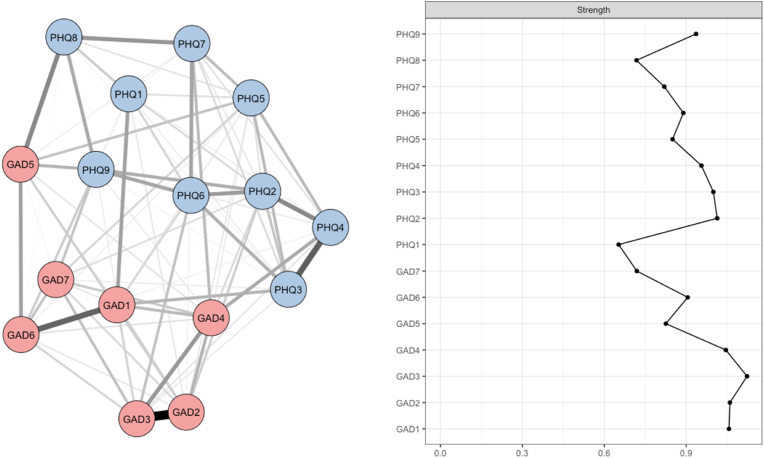
Network analysis at T2. **Legend:** The figure on the left displays the network analysis of depression (PHQ-9) and anxiety (GAD-7) symptoms at T2. The figure on the right shows strength centrality values for individual items of the PHQ-9 (depression) and GAD-7 (anxiety) scales. PHQ-9 items include: PHQ1 = Anhedonia, PHQ2 = Sad Mood, PHQ3 = Sleep, PHQ4 = Energy, PHQ5 = Appetite, PHQ6 = Worthlessness, PHQ7 = Concentration, PHQ8 = Psychomotor, PHQ9 = Suicidal Ideation; GAD-7 items include: GAD1 = Nervousness, GAD2 = Uncontrollable Worry, GAD3 = Excessive Worry, GAD4 = Trouble Relaxing, GAD5 = Restlessness, GAD6 = Irritability, GAD7 = Fear of Awful Events.

The analysis of relationships between PHQ-9 and GAD-7 symptoms at T2 reveals a network structure characterized by slightly reduced connectivity compared to T1, with 88 non-zero edges out of 120 possible. Despite this decrease, the average edge strength slightly increased, suggesting a general intensification of symptom associations (Fig. 3). The symptom network at T2 continues to reflect a relative separation between depressive and anxiety clusters, although certain connections suggest increasing overlap between these two clinical dimensions. Among the strongest associations, robust links were observed between GAD1 (Nervousness) and GAD2 (Uncontrollable), as well as between GAD3 (Excessive Worry) and GAD4 (Trouble Relaxing), indicating strong interrelations among anxiety symptoms. Similarly, key depressive symptom interactions, such as PHQ3 (Sleep) and PHQ4 (Energy), retained high weights within the overall network structure. Centrality analysis revealed notable shifts compared to T1. At T2, the symptoms with the highest strength centrality indices were GAD3 (Excessive Worry) (1.53), GAD2 (Uncontrollable Worry) (1.08), and GAD1 (Nervousness) (1.05), indicating a more prominent role for anxiety symptoms in the organization of the network. These results contrast with T1, where depressive symptoms occupied more central positions. This reorganization may reflect a shift in the symptom experience, with an increasing burden of anxiety among participants. The average edge weight was estimated at 0.23 (95 % CI [0.15–0.38]), confirming a slightly denser structure in the strongest connections (Supplementary Data, Fig. 5). Bootstrap analysis of network stability indicated satisfactory robustness, with a mean strength centrality coefficient of 0.516. Although slightly lower than at T1, this value still suggests a relatively stable symptom structure (Supplementary Data, Fig. 4). These findings suggest that the evolution from T1 to T2 is marked by a shift in central symptom nodes, with stronger links among anxiety symptoms. This structural change may reflect a transformation in the psychopathological dynamics of participants, in which anxiety plays an increasingly central role in the organization of depressive and anxiety symptoms.

**Fig. 4:**
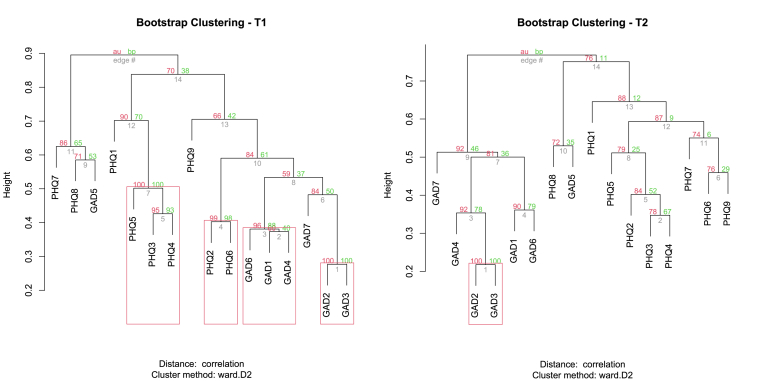
Edge stability in symptomatic networks at T1 and T2 **Legend:** The dendrograms represent the stability of the symptomatic network edges at T1 (left) and T2 (right), assessed by bootstrapping. Each branch corresponds to a group of symptomatic connections, with green values indicating the frequency of repetition (in %) in the simulated samples. Red boxes indicate stable groupings (clusters) with their confidence level. We observe an increase in link robustness at T2, suggesting a more coherent and consolidated organization of the symptomatic network.

**Fig. 5:**
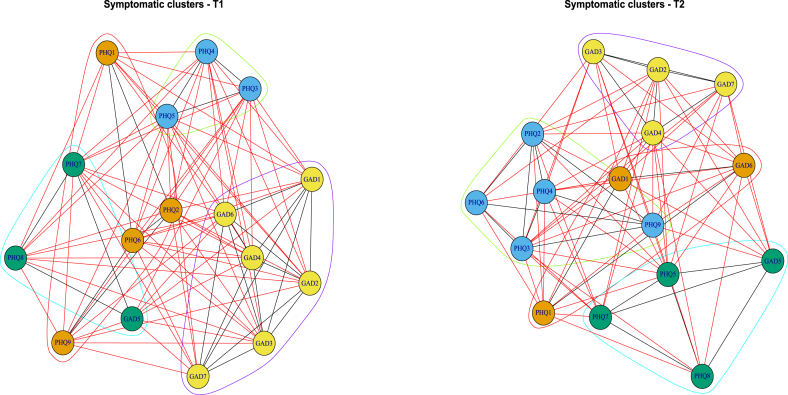
Evolution of the Symptom Network Structure from T1 to T2: Toward Increased Integration of Anxious and Depressive Symptoms **Legend:** On the left, the T1 network shows a relative separation between the anxious and depressive clusters. On the right, at T2, the two dimensions appear more integrated, suggesting a shift toward increased symptom co-occurrence. The colors indicate symptom communities automatically detected through clustering algorithms.

### 2.5 Evolution between T1 and T2

The evolution of the symptom network between T1 and T2 shows overall structural stability, as confirmed by the NCT p-value (p = 0.827), indicating no significant differences at the global level. However, localized changes were observed in specific symptom connections. The comparison of networks highlighted several edges whose strength significantly changed between T1 and T2 (Supplementary Data, Fig. 7). Among them, the connection between PHQ2 (Sad Mood) and PHQ4 (Energy) was strengthened (p = 0.024), as were the links between PHQ1 (Anhedonia) and GAD1 (Nervousness) (p = 0.022) and between PHQ5 (Appetite) and GAD5 (Restlessness) (p = 0.015). Conversely, some connections weakened over time, including GAD2–GAD5 (Uncontrollable Worry – Restlessness) (p = 0.037), GAD4–GAD6 (Trouble Relaxing – Irritability) (p = 0.018), and PHQ6–GAD7 (Worthlessness - Fear of Awful Events) (p = 0.021). Regarding centrality indices, the analysis revealed few significant differences between T1 and T2, with the notable exception of PHQ9 (Suicidal Ideation), which showed a significant change in its expected influence (p = 0.001). The evolution of PHQ9 (Suicidal Ideation) suggests a symptomatic shift, in which its role in activating other symptoms has diminished. This shift was accompanied by stronger links with anxiety-related symptoms such as GAD1(Nervousness) and GAD2 (Uncontrollable Worry), suggesting that suicidal ideation may increasingly align with an anxiety-based symptom profile rather than with classical depression. This shift toward greater centrality of anxiety symptoms may indicate a transformation in the psychopathological experience of participants, in which anxiety becomes a more dominant and structuring factor than depression (Supplementary Data, Fig. 6).

### 2.6 Cluster analysis

Bootstrap-based cluster analysis at T1 and T2 revealed an overall stable symptom structure, despite some noticeable changes. At T1, symptom groupings demonstrated strong coherence, with adjusted bootstrap probabilities (bp) exceeding 0.9 for most associations (Fig. 4). At T2, although several clusters reached maximal stability, a slight dispersion was observed within certain subclusters—particularly between anxiety and depressive symptoms—suggesting subtle reorganization of symptom interactions (Fig. 5). The approximated unbiased p-values (au) remained high (>0.8), confirming the robustness of clusters at T2. However, increased p-chis for certain edges indicated more pronounced variability, particularly around anxiety-related symptoms. Louvain visualizations also revealed greater overlap between depressive and anxiety clusters at T2 (Fig. 5). Moreover, suicidal ideation (PHQ-9) appeared more strongly connected to anxiety symptoms such as GAD5 (Restlessness) and GAD6 (Irritability). This trend suggests that suicidal ideation may progressively align with an anxiety-driven symptom dynamic rather than remaining strictly embedded within depressive processes. In summary, cluster analysis revealed overall structural stability, but also a greater intertwining of anxiety and depressive symptoms, with anxiety playing an increasingly central role in the organization of the psychopathological network (Fig. 4, Fig. 5). Taken together with the network and centrality analyses, these findings provide a nuanced understanding of the evolving symptom dynamics, both structurally and statistically.

## 3 Discussion

This study examined the evolution of anxiety and depressive symptoms among young adults experiencing socioeconomic precarity between 2020 (T1) and 2024–2025 (T2). The findings indicate a significant deterioration in participants’ mental health over time. Mean scores on the PHQ-9 and GAD-7 increased, and the proportion of individuals presenting with moderate to severe symptomatology also rose. The risk of suicidal ideation more than doubled (aOR = 2.16). Network analysis revealed a progressive transformation in symptom structure. While depression was more central at T1, anxiety symptoms became predominant at T2, particularly in their connection to PHQ-9 (suicidal ideation). Cluster analysis confirmed overall structural stability but also revealed reduced coherence in symptom groupings, suggesting increasing overlap between anxiety and depression. The Network Comparison Test indicated general structural invariance but identified important localized changes. These results suggest that anxiety symptoms may represent a key target for intervention in suicide risk prevention strategies, considering the dynamic evolution of psychopathological structures among young people facing socioeconomic hardship.

### 3.1 Comparison with the literature

The mental health trajectory of young adults during the COVID-19 pandemic has been characterized by a progressive deterioration, as demonstrated by several recent studies. Longitudinal research (Blackwell et al., 2024) has shown a sustained increase in anxiety and depressive symptoms among youth, particularly within socioeconomically disadvantaged populations. A study by Zhu et al. (Zhu et al., 2024) highlighted the persistence of anxiety disorders well beyond the acute phase of the pandemic, reflecting a chronic psychopathological dynamic fueled by structural uncertainty, socioeconomic insecurity, and loss of direction. Additionally, research by Yu et al. (Yu et al., 2024) identified excessive social media use and sleep disturbances as aggravating factors, especially among young adults. These effects are exacerbated in contexts of precarity, as shown by Adise et al. (Adise et al., 2024) whose work on socioeconomic inequalities revealed heightened exposure to psychological distress among young people facing residential, financial, and health instability.

Our findings are consistent with this body of literature but extend beyond the pandemic period. The significant increases in depression and anxiety scores, along with the rise in suicidal ideation between 2020 (T1) and 2024–2025 (T2), confirm a concerning deterioration in the mental health of socioeconomically vulnerable young adults. Symptom network analysis reveals a shift in centrality patterns: while depressive symptoms were dominant at T1, anxiety symptoms—particularly GAD3 (Excessive Worry), GAD2 (Uncontrollable Worry), and GAD1 (Nervousness), became more central at T2. This reorganization echoes the findings of Fried et al. (Fried et al., 2022), who describe a gradual shift in central symptoms toward anxiety, interpreted as an adaptive response to prolonged precarity and lack of future prospects.

In addition, our findings reveal an increasing entanglement between depressive and anxiety symptoms, as evidenced by the cluster analysis. This evolution is further supported by the strengthened links between anxiety symptoms and suicidal ideation, particularly through nodes GAD5 (Restlessness) and GAD6 (Irritability). This clinically concerning shift suggests that anxiety may no longer act solely as a worsening factor for depression, but rather as a direct contributor to suicide risk. This observation aligns with the findings of Xu et al. (Xu et al., 2024) and Bentley et al.(Bentley et al., 2016), and underscores the need for a paradigm shift in mental health policy, placing greater emphasis on anxiety disorders, which are often underestimated in suicide prevention strategies. This symptomatic shift also raises important questions regarding the underlying mechanisms and the heterogeneity of suicidal pathways in socioeconomically vulnerable populations. Traditionally conceptualized as a consequence of depressive hopelessness, suicidal ideation may also emerge through a distinct anxiety-based trajectory. Bentley et al. (2016) demonstrated that anxiety disorders are independent and significant predictors of suicidal thoughts and behaviors, even after controlling for depressive symptoms. Xu et al. (2024) showed that, in adolescents, anxiety symptoms, especially those related to physiological arousal and fear, were directly linked to suicidal ideation, bypassing depressive intermediaries. In our study, the increasing integration of PHQ9 (Suicidal ideation) within the anxiety cluster at T2 supports this model, suggesting a possible reorganization in the pathways leading to suicidality.

One plausible mechanism involves the concept of anxious hyperactivation (Ribeiro et al., 2011), where excessive physiological arousal (e.g., restlessness, insomnia, persistent worry) overwhelms cognitive and emotional regulation. Unlike depressive withdrawal, this dynamic may generate a sense of entrapment and loss of control, with suicide perceived as the only escape from intolerable inner tension. Björkenstam et al. (2024) also reported a sharp increase in anxiety symptoms in the weeks preceding suicide, highlighting their potential role as immediate prodromes. These findings suggest that interventions should not only address emotional dysregulation or depressive affect but must also specifically target the hyperarousal mechanisms inherent in anxiety-related suicidality. In sum, our results point to an increasingly salient anxiety–suicidality axis, warranting both theoretical reconsideration and adapted public health responses.

Despite the overall increase in symptoms, the Network Comparison Test did not reveal major structural changes in the symptom network between T1 and T2 (p = 0.827). This overall stability, coupled with localized changes in specific symptom connections, suggests a gradual reorganization of psychopathological dynamics rather than a structural rupture. Anxiety emerged as a more central symptom than depression in the T2 network, suggesting a shift in the structure of psychological distress over time. In the context of ongoing socioeconomic adversity, this evolution may reflect a gradual reorganization of symptom dynamics, in which anxiety becomes more prominent in the expression of emotional suffering. This observation aligns with previous studies reporting similar patterns of symptom centrality (Bai et al., 2021; Ochnik et al., 2024), Rather than interpreting anxiety solely as a transient response to acute stress, these findings suggest it may represent a more persistent and structured manifestation of distress in uncertain environments.

These findings are consistent with recent studies conducted in similar sociocultural contexts, including both general and student populations, which also highlight the growing centrality of anxiety symptoms in the aftermath of the pandemic. These findings are consistent with those of Bai et al. (Bai et al., 2021), who identified anxiety as a structuring factor in depression-anxiety comorbidity, challenging approaches focused exclusively on depressive symptoms. In contrast, our results diverge from those of Hoffart et al. (Hoffart et al., 2022), who attributed a central role to rumination in the maintenance of post-pandemic mental health disorders. Our findings instead support the notion of a functional adaptation to chronic and diffuse stress, in which anxiety becomes a dominant response pattern. This hypothesis is further supported by the study of Ochnik et al. (Ochnik et al., 2024) which documented a similar shift in symptom centrality among university students, from an initial dominance of anhedonia to the emergence of anxiety as social restrictions eased. In our sample, this phenomenon appears to be amplified by the cumulative effects of socioeconomic precarity. Although our study focused on young adults facing socioeconomic precarity, these findings may cautiously inform reflections on student populations, who are also increasingly exposed to uncertainty, precariousness, and mental health challenges. The recent study by Macalli et al. (Macalli et al., 2025) underscores a high prevalence of depression and suicidal ideation among university students in France, highlighting structural risk factors such as financial hardship, social isolation, and unequal access to psychological support. Thus, our results highlight an unstable yet structured symptom dynamic, in which anxiety emerges as a central element of psychological distress in socioeconomically vulnerable young adults. This configuration calls for a rethinking of screening and intervention strategies, with a stronger focus on anxiety symptoms, often minimized or overlooked in mental health policies that primarily target depression. Given the increasing centrality of anxiety and its direct association with suicidal ideation, these symptoms must now be considered priority targets for prevention and treatment, particularly in settings characterized by social vulnerability. Although the absence of longitudinal follow-up limits the analysis of individual trajectories, our repeated cross-sectional approach allows for the identification of structural changes at the population level. By comparing two independent samples collected four years apart, the study highlights the progressive reorganization of symptom networks in a context of persistent socioeconomic adversity. These findings underscore the relevance of context-sensitive mental health monitoring among vulnerable youth.

### 3.2 Theoretical and clinical Implications

The evolution of symptom networks between T1 and T2 reveals increased centrality of anxiety symptoms, particularly nervousness and difficulty relaxing. This symptom reconfiguration suggests that psychological interventions should move beyond the sole objective of reducing depressive symptoms and give specific attention to anxiety disorders. Traditionally focused on emotional regulation, current approaches would benefit from more fully integrating the real and contemporary concerns of young adults experiencing socioeconomic precarity. Rather than relying exclusively on stress management techniques, it seems essential to understand the deeper sources of their anxiety: feelings of insecurity, the ecological crisis, and economic and geopolitical uncertainty. In this context, “not feeling well” may be viewed less as a disorder to be corrected and more as a lucid response to an unstable environment.

Our findings underscore the need for integrative approaches that combine conventional therapeutic tools with close attention to the social context. The growing link between anxiety and suicidal ideation calls for a rethinking of suicide prevention frameworks to include strategies that address structural determinants of distress. In this regard, Guedeney et al. (Guedeney et al., 2023) demonstrated that a proactive offer of psychotherapy within local support systems, such as *Missions Locales*, significantly reduced suicidal ideation among young people in social integration programs. This highlights the value of situated, accessible interventions embedded in environments where young adults already seek support. Advancing research on the mechanisms that trigger anxiety, alongside protective factors such as health coverage, food security, housing stability, and community networks, remains crucial to designing effective, context-sensitive preventive strategies.

### 3.3 Limitations

While this study offers valuable insights into the evolution of symptom networks among socioeconomically vulnerable young adults, several methodological limitations must be acknowledged. The repeated cross-sectional design, although informative, does not allow for the tracking of individual trajectories or causal inference. Moreover, reliance on self-reported measures may introduce biases, particularly in a population facing multiple stressors that can influence symptom expression. Although the study controlled for basic sociodemographic variables, contextual factors such as perceived stress, trauma exposure, or major life events were not measured. This limitation may influence symptom expression and should be addressed in future longitudinal designs to better capture individual variability. Key contextual variables such as trauma exposure, substance use, or access to mental health care were not included and may have shaped the observed dynamics. Additionally, the sample drawn from a vocational support program, may not be representative of all young people in precarious situations, particularly those disconnected from institutional networks. Although network analysis provides a rich perspective on symptom structure, it remains correlational in nature and shifts in centrality should be interpreted with caution. Finally, the broader post-pandemic context marked by inflation, ecological anxiety, and ongoing social instability was not fully captured in our measures but likely influenced the participants’ psychological states.

## 4 Conclusion

This study highlights a concerning evolution in the mental health of socioeconomically vulnerable young adults between 2020 and 2024–2025, marked by significant increases in depressive and anxiety symptoms as well as suicidal ideation. Network analysis revealed a progressive shift in symptom centrality from depression to anxiety, suggesting a functional adjustment in the way young people respond to an unstable socioeconomic environment. One of the major contributions of this study lies in identifying anxiety not merely as a comorbid or aggravating factor, but as a structuring element of psychological distress and a direct predictor of suicide risk. This symptomatic shift calls for a rethinking of prevention and care strategies, with anxiety disorders fully integrated into targeted interventions, particularly for young people facing social vulnerability. Our findings also emphasize the need for integrated frameworks that combine psychotherapeutic interventions with responses to the social determinants of mental health. In a context where uncertainty has become structural, it is essential to broaden our understanding of psychological suffering beyond classical diagnostic categories, to include the economic, social, and political realities that weigh on younger generations.

## Ethical statment

This study was conducted in accordance with the principles outlined in the Declaration of Helsinki and adhered to all applicable institutional and national ethical guidelines. Ethical approval for the research protocol was obtained from the Ethics Committee of the University of Lorraine (AE2025-0037). Participants provided informed consent prior to their participation. Data were anonymized and handled in compliance with data protection regulations. No financial or personal conflict of interest influenced the conduct or reporting of this research.

## CRediT authorship contribution statement

**Aziz Essadek:** Writing – review & editing, Writing – original draft, Visualization, Validation, Methodology, Investigation, Funding acquisition, Formal analysis, Data curation, Conceptualization. **Tamara Guenoun:** Writing – review & editing, Writing – original draft, Visualization, Validation, Methodology, Conceptualization. **Florence Gressier:** Writing – review & editing, Writing – original draft, Visualization, Validation, Supervision. **Maha Najdini:** Writing – review & editing, Writing – original draft, Visualization. **Maud Cappelletti:** Writing – review & editing, Writing – original draft. **Antoine Frigaux:** Writing – review & editing, Writing – original draft. **Maria Melchior:** Writing – review & editing, Writing – original draft, Validation. **Maeva Musso:** Writing – review & editing, Writing – original draft, Visualization, Validation. **Marion Robin:** Writing – review & editing, Writing – original draft, Visualization, Validation, Supervision, Software, Resources.

## Ethical approval

This research was approved by ethics committees. All relevant information has been included in the article.

## Funding source

Aziz Essadek was funded by the Fondation Hospitalière pour la Recherche sur la Précarité et l’Exclusion sociale (grant no. 2022-04042).

## Conflict-of-interest statement

All authors declare that they have no conflicts of interest.

## Data Availability

Data will be made available on request.
