# Supplementary material for: Post-pandemic changes in anxiety and depression symptom networks among socioeconomically disadvantaged young Adults: A repeated cross-sectional study
Source: SSM Popul Health. 2025 Aug 16;31:101854. doi: 10.1016/j.ssmph.2025.101854 (PMC12398219; doi:10.1016/j.ssmph.2025.101854)
Supplement: Multimedia component 1 [file mmc1.pdf]

Supplementary Data

Table 1 : Characteristics of the sample

Table 2 : Odds Ratios and Adjusted Odds Ratios (T1 vs T2)

Figure 1 : Correlation matrix after npn transformation T1

Figure 2 : Correlation matrix after npn transformation T2

Figure 3 : Comparison between observed and bootstrapped edge weights in the symptom network T1

Figure 4 : Correlation matrix of node strength centrality between PHQ-9 and GAD-7 items T1

Figure 5 : Comparison between observed and bootstrapped edge weights in the symptom network T2

Figure 6 : Correlation matrix of node strength centrality between PHQ-9 and GAD-7 items T2

Table 1: Characteristics of the sample

Characteristic	T1 Total N= 960 n (%) or Mean [SD]	T2 Total N = 380 n (%) or Mean [SD]	Statistics	Effect size
Age	21.2 [2.1]	21.5 [2.1]	T = -2.15 ; P=0.032	-0.13[a]
Gender				
Female	475 (49.5)	189 (49.7)	Chi-2 = 7.64 : p=0.022	0.07[b]
Level				
Higher than baccalaureate	90 (9.4)	77 (20.3)	Chi-2 = 29.75 : p<0.001	0.15[b]
Baccalaureate	319 (33.2)	107 (28.2)		
Lower than baccalaureate	551 (57.4)	196 (51.6)		
Accommodation				
Family	591 (61.6)	(61.05)	Chi-2=10.25, p=0.017	0.08[b]
Tenant	136 (14.2)	76 (20)		
Emergency shelter	155 (16.1)	43 (11.32)		
Homeless	78 (8.12)	29 (7.63)		
Live Alone				
Yes	310 (32.3)	133 (35)	Chi-2=0.78, p=0.376	
Financial assistance				
Yes	225 (23.4)	81 (21.3)	Chi-2=0.58, p=0.446	
Jobless				
Yes	744 (77.5)	152 (77.2)	Chi-2=0.25, p=0.615	
Bourse				
Yes	225 (23.44)	81 (21.32)	Chi-2=0.58, p=0.446	
Financial resource				
No resource	465 (48.4)	186 (48.95)	Chi-2=21.32, p<0.001	0.13[b]
<500 Euros	271 (28.2)	70 (18.42)		
>500 et < 1000 Euros	153 (15.9)	93 (24.47)		
> à 1000 Euros	71 (7.4)	31 (8.16)		
**Psychiatric symptoms**				
PHQ-9	9.43 [6.7]	11.35 (7.2)	T = -4.47 ; p<0.001	
GAD-7	6.3 [5.9]	8.14 (6.2)	T = -4.95 ; p<0.001	
**Psychiatric symptoms with cut-offs**				
PHQ-9 ≥ 10	450 (46.9)	214 (56.3)	Chi-2=9.33, p=0.002	
GAD-7 ≥ 8	352 (36.7)	182 (47.9)	Chi-2=13.85, p<0.001	
Suicidal ideation	249 (25.9)	163 (42.9)	Chi-2=35.97, p<0.001	

Legend: Abbreviation: PHQ-9, 9-item Patient Health Questionnaire; GAD-7, 7-item Generalized Anxiety Disorder.
a) Cohen's d, b) Cramer's V

Table 2 : Odds Ratios and Adjusted Odds Ratios (T1 vs T2)

Outcome	OR	p-value	Adjusted OR 95% CI	p-value
PHQ (Depression)	1.46 [1.15-1.85]	p=0.002	1.46 [1.14 – 1.87]	p=0.003
GAD (Anxiety)	1.59 [1.25-2.02]	p<0.001	1.54 [1.20 – 1.97]	p<0.001
Suicide	2.15 [1.67-2.75]	p<0.001	2.16 [1.67 – 2.81]	p<0.001

Legend: This document presents the adjusted odds ratios (OR) for depression (PHQ), anxiety (GAD), and suicidal ideation between T1 and T2, along with their 95% confidence intervals (CI) and statistical significance (p-values). Adjustments were made for age, gender, education level, housing type and income level.

Figure 1 : Correlation matrix after npn transformation T1

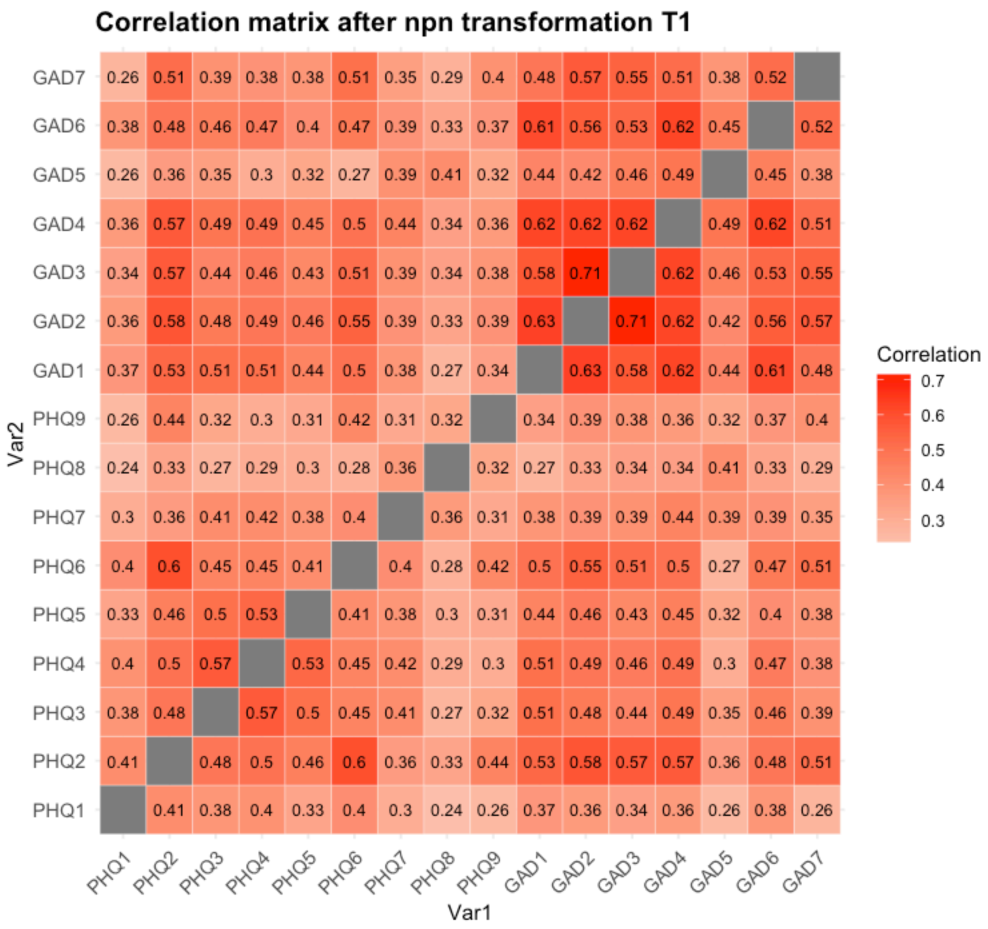

Figure 2 : Correlation matrix after npn transformation T2

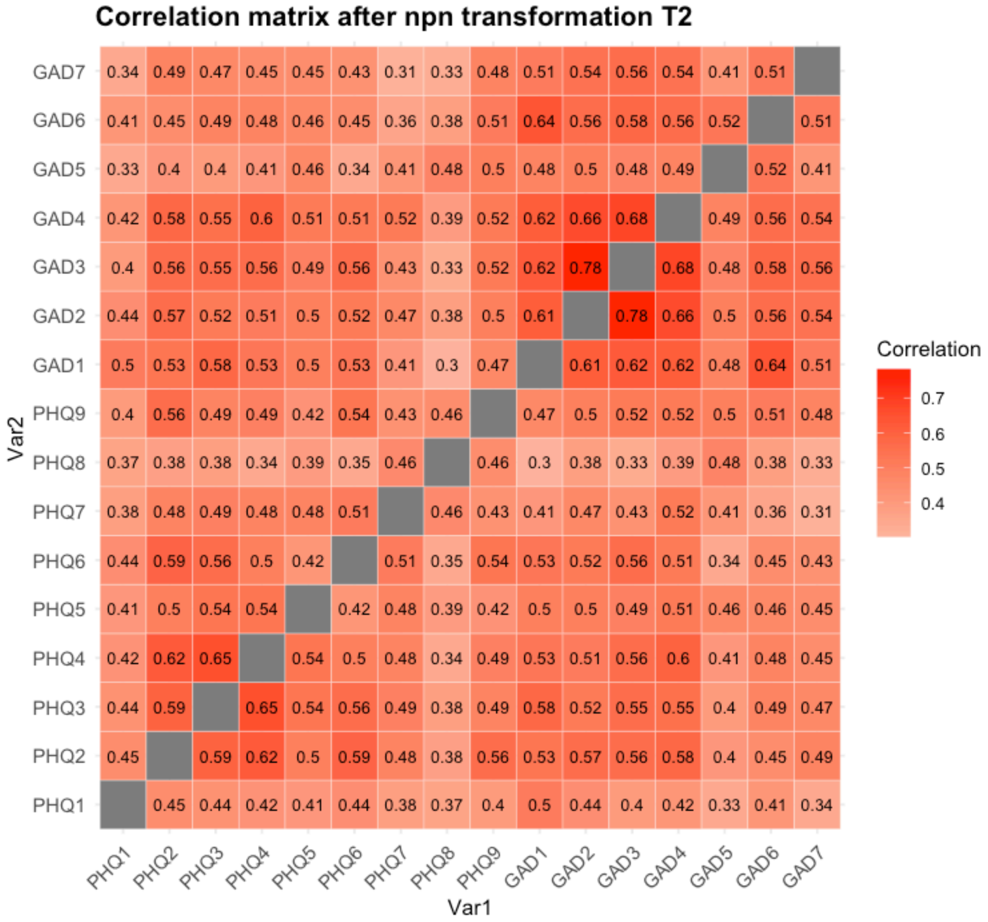

Figure 3: Stability of the centrality indices (strength).

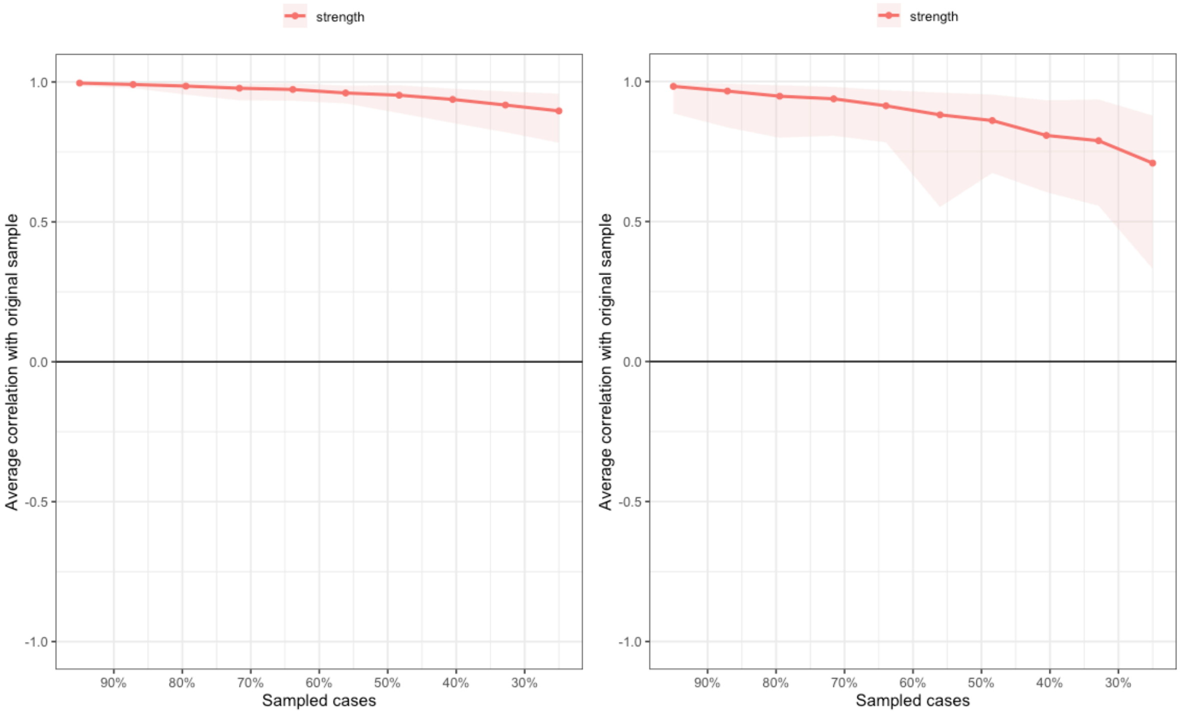

<u>Legend:</u> The figure on the left represents strength at T1, and the figure on the right at T2.

Figure 4 : Comparison between observed and bootstrapped edge weights in the symptom network T1

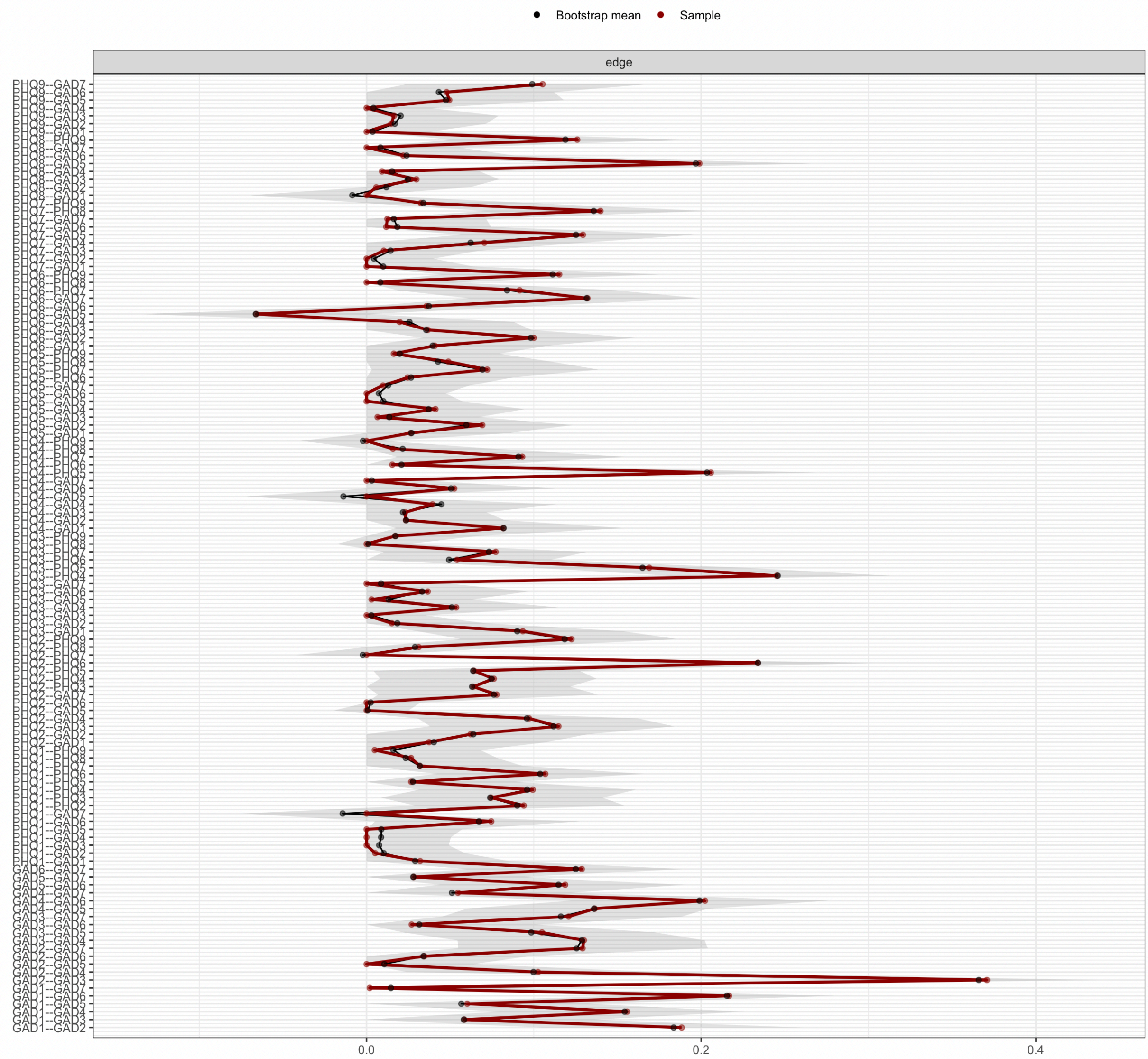

<u>Legend :</u> The plot displays the differences between edge weights estimated in the original sample (red dots) and the bootstrap means (black dots), with confidence intervals. This visualization assesses the stability of connections between PHQ-9 and GAD-7 symptoms.

Figure 5 : Correlation matrix of node strength centrality between PHQ-9 and GAD-7 items T1

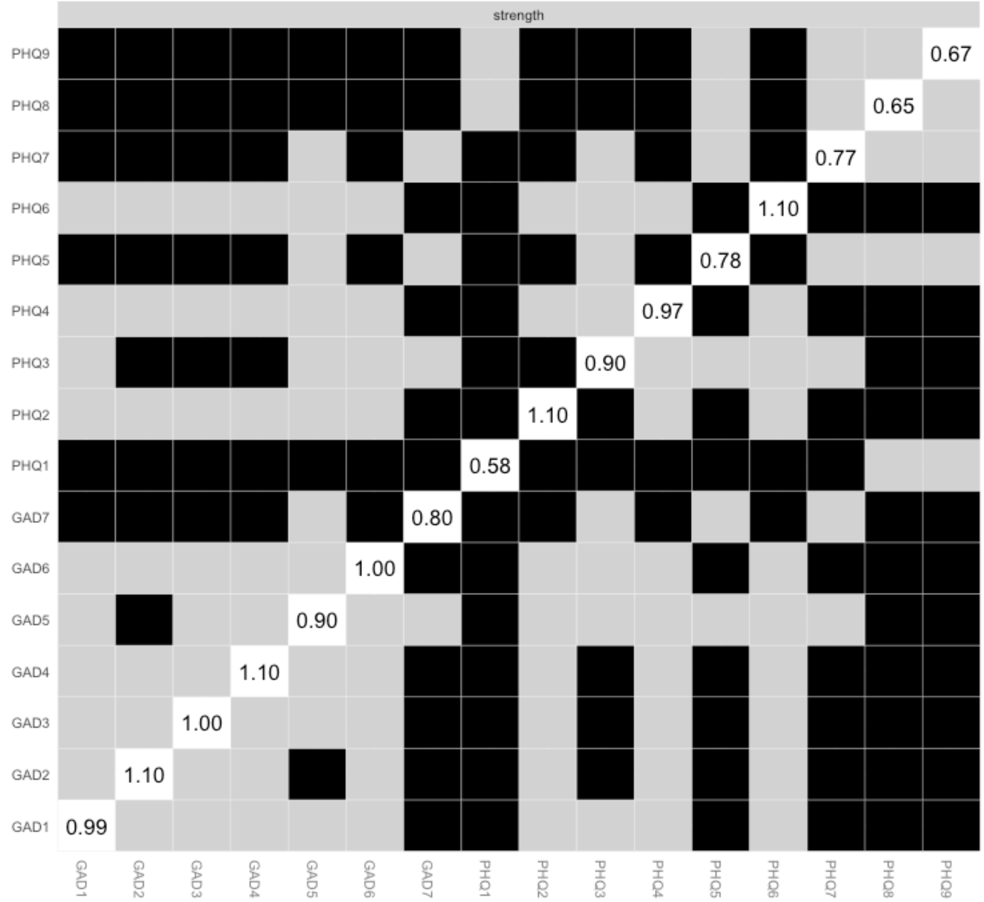

<u>Legend :</u> The matrix shows correlations between strength centrality scores for each item pair. Numeric values indicate correlation coefficients; black squares represent low or non-significant correlations.

Figure 6 : Comparison between observed and bootstrapped edge weights in the symptom network T2

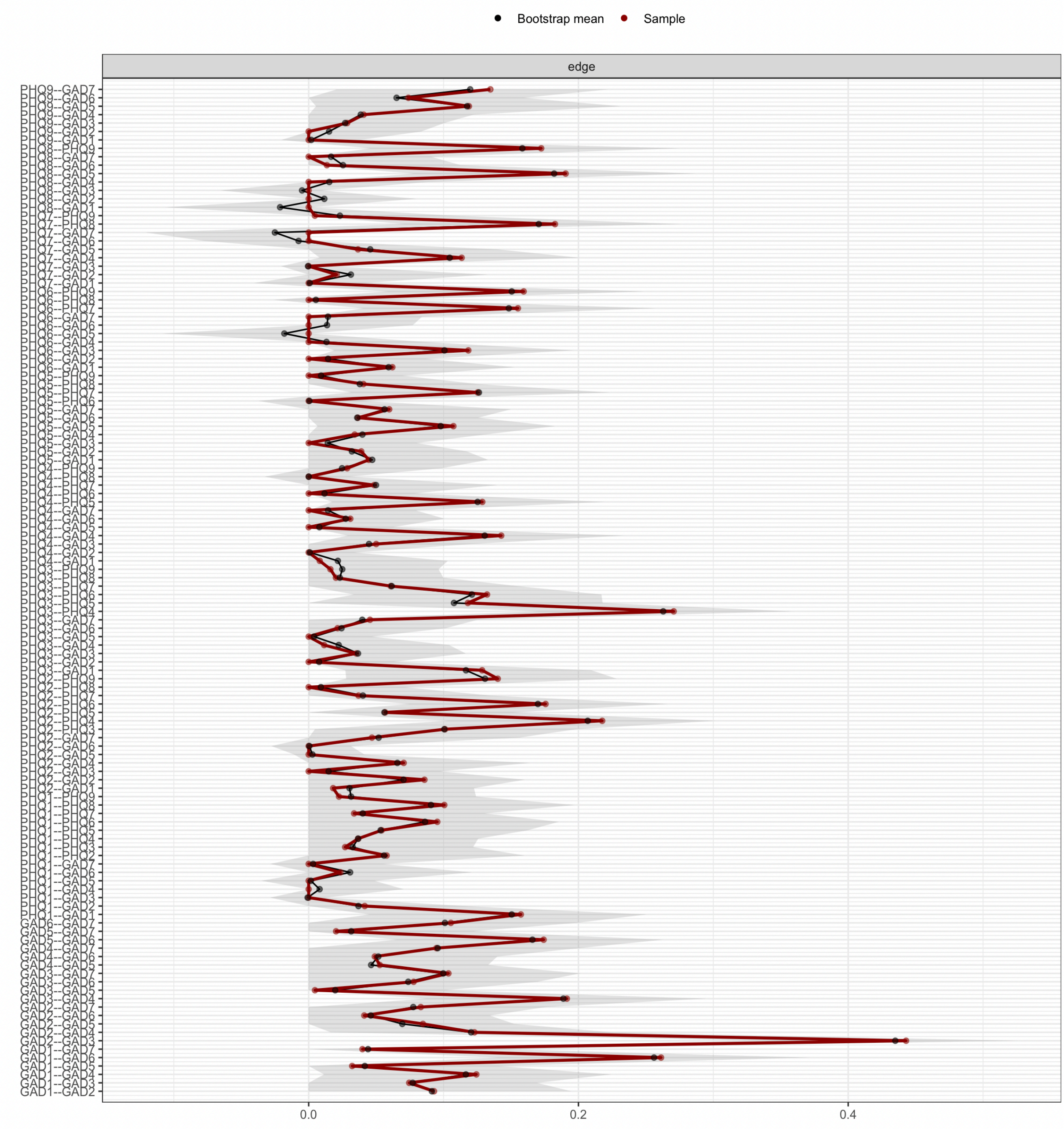

<u>Legend :</u> The plot displays the differences between edge weights estimated in the original sample (red dots) and the bootstrap means (black dots), with confidence intervals. This visualization assesses the stability of connections between PHQ-9 and GAD-7 symptoms.

Figure 7 : Correlation matrix of node strength centrality between PHQ-9 and GAD-7 items T2

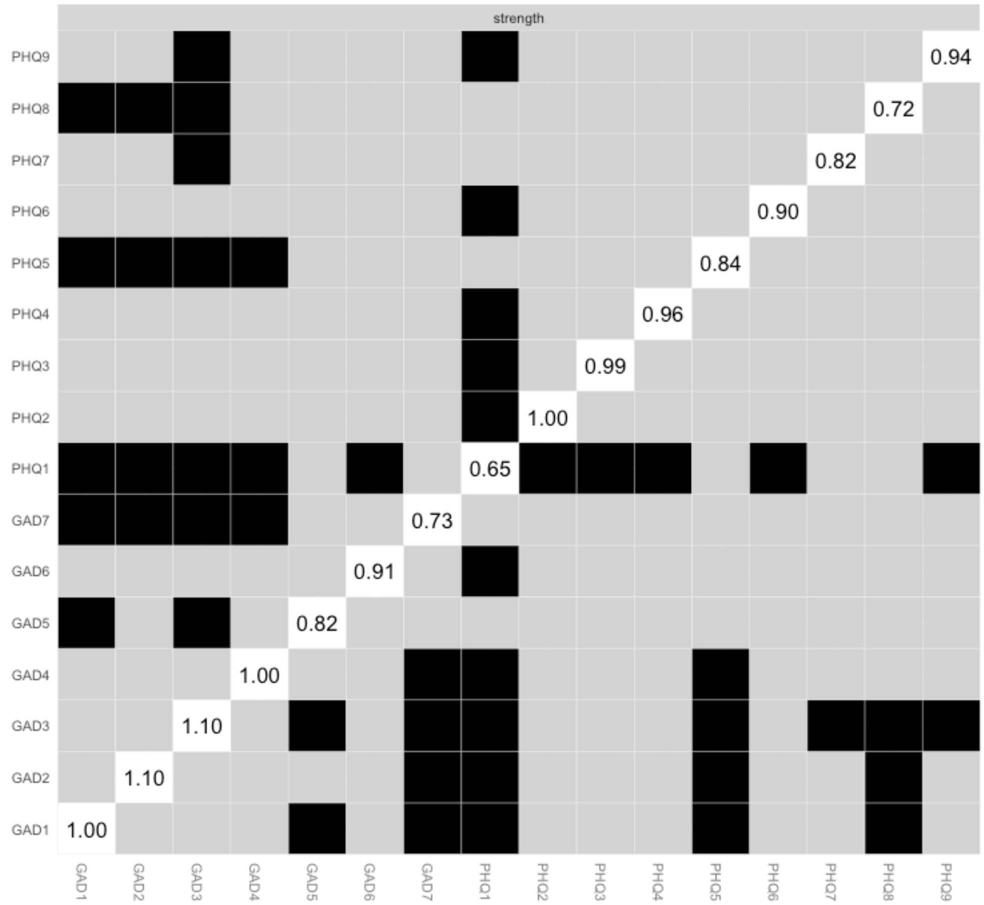

<u>Legend :</u> The matrix shows correlations between strength centrality scores for each item pair. Numeric values indicate correlation coefficients; black squares represent low or non-significant correlations.
